# Association between Neighborhood Food Access, Household Income, and Purchase of Snacks and Beverages in the United States

**DOI:** 10.3390/ijerph17207517

**Published:** 2020-10-15

**Authors:** Ke Peng, Nikhil Kaza

**Affiliations:** 1 Department of Urban Planning, School of Architecture, Hunan University, Changsha 410082, China; 2 Department of City and Regional Planning, University of North Carolina at Chapel Hill, Campus Box 3140, Chapel Hill, NC 27599, USA; nkaza@unc.edu

**Keywords:** food availability, convenience stores, supermarkets, region, accessibility, street connectivity, diversity

## Abstract

Considerable research on the risk factors of obesity and chronic diseases has focused on relationships between where people live, where they shop, and the types of food they purchase. Rarely have investigators used a national sample and explicitly addressed the amount of energy-dense and nutrient-poor foods purchased in different types of neighborhood food stores. Even more rarely have studies accounted for the characteristics of the broader built environment in which food stores are located and which affect the convenience of using neighborhood food stores. We used a large population-based cohort of predominantly white U.S. households from the Nielsen Homescan Consumer Panel 2010 dataset to examine whether there were positive cross-sectional associations between availability of neighborhood convenience stores and supermarkets and self-reported household annual expenditures for snacks and beverages. We examined this relationship separately for poor and non-poor households as defined by the 2010 U.S. federal poverty threshold. We used mixed error-component regression models to examine associations between availability of neighborhood food stores and the expenditures on snacks and beverages, controlling for regional destination accessibility, availability and diversity of neighborhood destinations, and neighborhood street connectivity. In multivariate analyses, we observed that poor households in neighborhoods with few convenience stores purchased more snacks than poor households in neighborhoods with many convenience stores (b = −0.008, *p* < 0.05). Non-poor households in neighborhoods with many convenience stores and fewer supermarkets purchased more snacks than non-poor households in neighborhoods with few convenience stores and many supermarkets (b = 0.002, *p* < 0.05 for convenience stores; b = −0.027, *p* < 0.05 for supermarkets). Increase in number of convenience stores decreased the purchase of snacks by poor households, but increased in non-poor households. On other hand, increase in number of supermarkets discouraged purchase of snacks by non-poor households but had no effect on the purchasing behavior of the poor-households.Therefore, evaluation of access to energy-dense and nutrient-poor foods should include a consideration of geographic proximity. Local governments should consider strategies to expand the availability and access to nutrient-rich food and beverage products in convenience stores for consumers.

## 1. Introduction

Since realizing that food and the broader built environment are related to the risk of incurring chronic diseases [1,2], investigators have conducted many studies on food access. A majority of this research [3,4,5,6,7] has examined the access to fresh vegetables and fruits. There is limited literature on access to energy-dense and nutrient-poor (EDNP) foods, such as sugary beverages and sweet snacks. Academics have been concerned about the negative effects of EDNP foods on individual health [8,9,10]. We can better understand access to food by studying how neighborhood food access affects purchases of EDNP foods. The food purchasing literature provides, at most, moderate evidence that neighborhood food availability relates to the amount of fresh foods purchased or the food purchased as a total based on an aggregated purchasing index [6,11,12,13]. Because of the ubiquity of EDNP foods and their relatively low cost [14,15], it is not known whether people rely on the immediate neighborhood to purchase EDNP foods.

Convenience stores accounted for the largest proportion of retail food outlets [16], which constitute a nonignorable outlet for the purchase of EDNP foods, compared with other types of small food stores such as pharmacies, dollar stores, and small grocery stores. In the US, convenience stores are frequently located closer to people compared with larger food stores such as full-service supermarkets [16,17] and convenience stores sell mainly EDNP foods compared with full-service supermarkets. The spatial proximity of convenience stores and the dominant type of foods they sell may prompt people to buy relatively more EDNP food in such stores [18,19]. Several investigators have highlighted the health implication of convenience stores by observing that neighborhood convenience stores decreased residents’ diet quality [19,20,21,22]. However, generally, research that has addressed the relationship between availability of convenience stores and how people use those stores has been based on small sample sizes or targeted to subgroups or a single city or region. For example, from fifty Mexican households in Texas, Sharkey et al. [19] observed that people purchased larger amounts of total energy from foods and beverages with decreasing distance between home and the nearest convenience store. Sanders-Jackson et al. [18] estimated that more than four million U.S. adolescents used convenience stores once a week or more frequently, particularly African-Americans and individuals who lived in non-dense census tracts or areas of relative impoverishment. These estimates based on small sample size or a single area have weak generalizability to an entire population or to other subgroups. As well, although the supermarket offers more nutritious food than that offered by convenience stores, supermarkets, nevertheless, also devote more shelf space to EDNP foods than to nutritious food [23]. The influence of supermarkets on attracting EDNP food purchase has always been overlooked in previous studies [24,25,26].

Understanding differences between poor and non-poor (as defined by the U.S. federal poverty threshold) persons in food choice, i.e., EDNP foods versus more nutritious foods, helps to understand food access. Poor individuals appear to rely more on the immediate neighborhood to obtain food because of limited travel mobility and budget constraints. Convenience stores are closer to home than full-service supermarkets, and the dominant foods in convenience stores were EDNP foods [16]. In addition, poor households had relatively more convenience stores and fewer full-service supermarkets nearby compared with their non-poor counterparts [27,28]. Particularly because EDNP foods are normally cheaper than nutritious foods [14,15], the low-price enticement may make EDNP foods more affordable to poor individuals and families [29]. Therefore, it is possible that poor persons purchase more EDNP foods in neighborhoods with many convenience stores. Nonetheless, some literature implies that there is little difference between income groups in purchasing EDNP foods [30,31]. A less well-off person may travel far to buy junk food because it is easy to store and available in bulk. The cost in terms of time is greater for an affluent individual than for a less well-off person; thus, poor and non-poor persons may prefer to save time (for cooking and travelling) by buying EDNP foods. In sum, income may or may not influence purchasing patterns based on the presence of nearby food resources.

Although we focus on the proximity of food stores in this study, geographic proximity is just one of many forces that shape human behavior [32]. Some investigators have been concerned about the social environment that relates to food shopping that has been rarely addressed by food purchasing studies, such as juggling work and family responsibilities and interactions with shop personnel and proprietors [22,23]. We share this concern of social environment by explicitly examining aspects of the broader built environment that shape a household’s purchasing decisions. Those aspects included the availability and mix of neighborhood food and non-food destinations, opportunities for transportation, and social factors such as purchasing power and potential for ride-sharing at the neighborhood level. By including these issues, we partly address the potential interactions between households and the built and social environment that may increase or decrease the chances of purchasing EDNP foods.

We provide a nationwide continental United States study of the cross-sectional relationships between the presence of neighborhood convenience stores and supermarkets and purchases of snacks and beverages for 2292 poor households and 46,332 non-poor households in 378 Core Based Statistical Areas (abbreviated as CBSA below). Our objective was to examine whether there were associations between neighborhood availability of convenience stores and supermarkets and the purchase of small-sized, often nonnutritive food items by households in the two income brackets. This goal is intended to inform public policymakers to propose different strategies for convenience stores versus full-service supermarkets in promoting quality diet in the continental U.S. [15]. Our research is unique because we used a large population-based cohort of U.S. households and included other factors to quantitatively control for the broader built and social environment in which the food stores resided. Thus, we expected that the associations between neighborhood food availability and snacks and beverages purchased may be confounded less by incorporating other spatial and non-spatial factors that affect food purchasing behavior. Therefore, using a population-based sample, we eliminate a gap in understanding whether increasing the numbers of convenience stores and supermarkets in the immediate neighborhood encourages the purchase of small-sized, often nonnutritive food items. Our hypotheses are the following:

(1) Compared with households who lived in neighborhoods with few convenience stores, households in neighborhoods with many convenience stores had higher expenditures for snacks and beverages, regardless of whether the households were below or above the poverty threshold.
(2) Compared with households who lived in neighborhoods with few supermarkets, households in neighborhoods with more supermarkets had higher expenditures for snacks and beverages, regardless of whether the households were below or above the poverty threshold.

## 2. Materials and Methods

### 2.1. Study Sample

This report is a companion to our published article, “Availability of neighborhood supermarkets and convenience stores, broader built environment context, and the purchase of fruits and vegetables in U.S. households” [33]. Similar to the published article, we used the Nielsen Homescan Consumer Panel Dataset (abbreviated as Nielsen data) for 2010 [34], a nationwide collection of food and beverage purchase data from 40,000–60,000 United States households. Although Nielsen data did not disclose the type of store in which the foods were purchased, the Nielsen Consumer Panel Dataset Manual mentioned that the foods purchased were from four types of stores: food store (for example, grocery stores and supermarkets), warehouse club, drug store, and convenience store [34]. Nielsen households did not report purchases from restaurants, fast food places, or mobile vendors.

Unlike the published article that focused on packaged fresh fruits and vegetables by 20,000 households, we examined a larger sample (over 40,000) of snack and beverage purchases. From the 2010 Nielsen data (analyzed in 2018) for 60,658 households, we selected 60,239 that reported purchases of snacks or beverages. Our final sample was 48,624 households after we excluded households outside the CBSAs and households that lacked covariate information. We divided the 48,624 households into, respectively, 2292 poor (below the poverty line) and 46,332 non-poor (equal to or above the poverty line) households according to the 2010 federal poverty threshold from the U.S. Census Bureau, which considers the number of adults, the number of related children aged <18 years, and age. For example, the poverty line for a two-person, no-child household (age of household head <65) was USD 13,180, whereas the line for a two-person, one-child household (age of household head <65) was USD 14,973. Approximately 89% of poor households had an annual income below USD 20,000, compared with 5.4% of non-poor households. None of the poor households had an annual income greater than USD 60,000 compared with 58.7% of non-poor households.

### 2.2. Measures

#### 2.2.1. Purchases of Snacks and Beverages

Following the classification method by Pasquale et al. [20], we defined snacks as potato chips, tortilla chips, pork rinds, caramel corn, pretzel and similar items. We defined beverages as soft drinks, fruit drinks, and fruit juices. See Table S1 in Supplementary Material for details of product items purchased. We included fruit juice because of its high sugar content [35]. Fruit juice was increasingly seen as having negative health effects similar to sugar-sweetened beverages [36] because fructose consumption from the sucrose (even in 100% fruit juice) may be associated with liver injury and metabolic syndromes [37]. Nielsen data categorized the food products by product module, which indicated the specific type of food purchased. For example, we can know how much a household spent on potato chips because the product module indicated that the household purchased “snacks—potato chips”. Supplementary Material presents procedures for calculating the amounts of payment for snacks and beverages by Nielsen households. Nielsen households self-reported their purchases of packaged foods in retail stores through scanning the barcodes of the products they purchased (scanners provided by Nielsen) and recorded the number of each item purchased [38]. We calculated the purchases of individual households during the entire 2010 year to mitigate against anomalous purchasing behavior [38].

#### 2.2.2. Neighborhood Food Availability

We used the numbers of convenience stores and supermarkets in a 3-km buffer (abbreviated as neighborhood) around a residence to represent neighborhood food availability. We used ReferenceUSA 2010, a business information dataset, to retrieve convenience stores and supermarkets. We used primary 6-digit Standard Industrial Classification (SIC) codes from ReferenceUSA to classify convenience stores and supermarkets, following the definition of Rummo et al. [20] and our previous work [33]. Convenience stores were stores detached or attached to gas stations (SIC = 541,103, 554,100, and 554,199), variety stores (SIC = 533,100), and stores that sold snack products (SIC = 541,102). Supermarkets (SIC = 541,101) were chain or non-chain hypermarkets (greater than 9290 square meters), supermarkets (6132–9197 square meters), and superstores (5110–6039 square meters). The details of classifying food stores using SIC codes have been described [33]. Because Nielsen did not disclose the exact residential location of households, we used the geographic center of a household’s residential Zip Code Tabulation Area (ZCTA) to proxy Nielsen’s residential location.

#### 2.2.3. Covariates

Like our earlier study [33], we used features of built environment, such as regional destination accessibility, availability of neighborhood destinations (number of six types of food and non-food destinations in the 3-km buffer), neighborhood destination diversity (an entropy index calculated by using the six types of food and non-food destinations in the 3-km buffer), neighborhood street connectivity (road density in the 3-km buffer), and urbancity (urbanized area, urban cluster, and non-urban area). We used regional destination accessibility to account for the magnitude of chances that households may have purchased snacks and beverages in a broad activity space beyond the residential neighborhood. Although we were not able to know the true number of “exact food and non-food locations” that households frequented in the large area, a second-hand databased (namely, Smart Location Database) disclosed the total number of employees in a 45-min automobile travel from the centroid of Census Block Group in the U.S. We assumed that the number of employees in a 45-min automobile travel was a reasonable proxy for the size of potential destinations that households may access in the region, or the attractiveness in the region. The other built environment covariates were used to control for multi-purpose travel and the travel cost that may increase or decrease the likelihood of purchasing snacks and beverages in the neighborhood [39]. These covariates included availability and diversity of neighborhood destinations and neighborhood street connectivity. In calculating the availability and diversity of neighborhood destinations, we included three types of destinations visited before or after food shopping, which were other store/mall (department stores, retail shops and wholesale clubs), locations for socializing (childcare services, health-care services and churches), and restaurants (sit-down restaurants and fast food restaurants). We employed the American Time Use Survey 2010 to identify the major destinations that people frequent before and after food shopping. We used entropy value to calculate diversity of neighborhood destinations based on the three types of aforesaid destinations. We retrieved the road density value from the Smart Location Database to proxy neighborhood street connectivity. We calculated the variable of neighborhood street connectivity by averaging the road density values of block groups with centroids in the 3-km buffer. Because poor households may access food stores with assistance from neighbors (for example, by ride-sharing), we included neighborhood-level social environment variables of the percent of households that did not own a car and the percent of households below the poverty line. The Census Bureau developed such variables at either the residential census block group or tract level. The measures of these physical and social environment features at the neighborhood-level, region-level, and area-level characteristics were calculated based on the geographic center of residential ZCTA. Like our published article, we used individual and family-level socioeconomic attributes to control for the factors that affected household food purchase. We used six categorical variables (as self-reported by Nielsen households): female head of household education level, race identity, number of household members, marital status of household head(s), children, and number of employed household members. The detailed measures of these variables have been described [33].

### 2.3. Statistical Analyses

Nielsen data disclosed the ZCTA and CBSA codes in which households resided. The number of sampled Nielsen households within the same ZCTA ranged from 1 to 45 with a median value of three and an interquartile range equal to five; the number of sampled ZCTAs within the same CSBA ranged from 2 to 641 with a median value of 13 and an interquartile range equal to 18. Households in the same ZCTA or CBSA had many similarities that may have violated the principal of independently and identically distributed observations. To address the “multi-level” feature of the data, we implemented mixed error-component regression models for snacks and beverages. We used logarithmic transformations of the dependent variables (aka, food expenditures) in the mixed error-component regression models to account for the right-skewed distribution of outlays for snacks and beverages. The exposure variables were numbers of neighborhood convenience stores and supermarkets. Other independent variables were built environment features, the household-, and neighborhood-level sociodemographic covariates and expenditures (log-transformed) for beverages (in snack models) and expenditures (log-transformed) for snacks (in beverage models). We used lme4 package in R × 64 3.5.1 [40] to run mixed error-component regression models.

### 2.4. Sensitivity Analyses

Nielsen data did not disclose the exact home addresses of sampled households. Therefore, we considered that the geographic center of ZCTA may not have represented the actual residences of households and the center of ZCTA failed to describe the food and built environment based on such a “pseudo-residence”. For example, we may have underestimated the number of supermarkets in the neighborhood because we did not observe a supermarket in the 3-km buffer for over 70% of the households. To partially address this issue, we modeled a reduced sample that excluded households located in those extensive ZCTAs (>153.5 km^2^). In addition to 3 km, we used a larger buffer (5 km) to test the neighborhood effect of food and built environment. We used the 5-km buffer to calculate the numbers of convenience stores and supermarkets, availability and diversity of neighborhood destinations, and neighborhood street connectivity. The calculation of regional destination accessibility was fixed as a 45-min automobile drive from residence, which remained the same regardless of the buffer size. The conclusions of Kerr [41] et al. and Liu et al. [21] supported our assumption that a 3-km buffer may be too small to reflect the extent to which households prefer to access food stores, particularly large food stores.

## 3. Results

### 3.1. Descriptive Statistics

Only approximately 18% (8703 out of 48,624) of households in the sample were non-white, and two-thirds of the households’ female heads had an education level above high school (Table 1). Poor households had significantly different sociodemographic features, such as a lower percent with an educational attainment of high school or above, White, and a higher percent with children (Table 1).

Poor households purchased fewer snacks and beverages, when compared to non-poor households (Table 2). On average, Nielsen households had three convenience stores in their neighborhoods. Approximately 73% (35,301 out of 48,624) of the households did not have a supermarket in the neighborhood (Table 2). The median numbers of convenience stores for poor and non-poor households were both three; the percent of having no supermarket in the neighborhood for poor and non-poor households was 71.5 (1639 out of 2292) and 72.7 (33,683 out of 46,332), respectively. Poor and non-poor households differed significantly (*p* < 0.05) but not greatly in the numbers of convenience stores and supermarkets in their neighborhoods (Table 2). Poor households had a noticeably lower level of regional destination accessibility compared with their non-poor counterparts, although the differences in other built environment characteristics, such as availability and diversity of neighborhood destinations, were small and could be ignored. A reasonably high percent of poor households lacked automobiles; these households lived in neighborhoods with a higher percent of the population below poverty level and a higher percent living in non-urban areas, when compared to their non-poor counterparts. The CBSAs with a high percent (above nine and below seventeen) of poor households and those with a low percent (below three) were distributed evenly in the United States (Figure S1 in Supplementary Material). Eleven percent (43 out 378) of CBSAs did not sample poor households, such as Abilene, TX, Ann Arbor, MI, and Beckley, WV. The CBSA with the highest percent (18.2%) of poor households was Brunswick, GA.

### 3.2. Regression Analyses

Table 3 and Table 4 present the results of regression analyses. For the poor households, we found that they purchased significantly fewer (by 0.008 × 100% = 0.8%, *p* < 0.05) snacks for each one-convenience-store increase in a neighborhood (Table 3). With increases in the numbers of supermarkets from zero to non-zero, expenditures on snacks did not differ significantly. Non-poor households obtained more (by 0.002 × 100% = 0.2%, *p* < 0.05) snacks for each one-convenience-store increase in the neighborhood. Non-poor households in a neighborhood with one supermarket acquired significantly fewer (by 0.027 × 100% = 2.7%, *p* < 0.05) snacks compared with non-poor households in neighborhoods without a supermarket (Table 3). With an increase in numbers of supermarkets or convenience stores, poor and non-poor households did not differ in beverage expenditures (Table 4).

Tables S2–S5 in Supplementary material present the results of sensitivity analyses, which produced results of similar magnitude, direction, and statistical significance. The major exception was that, for non-poor households, the expenditures on snacks increased with an increase in number of neighborhood convenience stores in the major sample but not in the reduced sample (aka, households exclusive to those that resided in large ZCTAs) or when we used a 5-km buffer to measure availability of neighborhood supermarkets and convenience stores. Some studies in the US. observed that 800–1000 m was used to capture convenience stores within peoples’ immediate reach in urban settings [17,42], and it was relatively easy to access EDNP foods such as soda within 3000 m in non-rural areas [43]. Therefore, we chose finally to report the results the of 3-km buffer.

## 4. Discussion

We found positive associations between neighborhood convenience store availability and snack purchase among non-poor households. The 0.8% increase in purchased snacks associated with an increase in one convenience store was not a trivial issue because there were 15% (6746 out of 46,332) and 4% (1653 out of 46,332) of non-poor households that had more than ten and twenty convenience stores in the 3-km buffer, respectively. The snacks purchased by households with a large number of convenience stores nearby were higher than purchases made by households with few convenience stores nearby. 

By using a national sample, we have expanded findings from single-city case studies which found that convenience stores in a small area were an accessible source of nutrient-poor foods [22,44]. Similarly, Liu et al. [17] and Shannon [45] reported on GPS-based studies in which people still relied on the food stores (for example, convenience stores and fast food restaurants) near their residences, although they purchased from full-service supermarkets and sit-down restaurants elsewhere. Our results are consistent with these GPS-based studies because they suggest that many convenience stores within a 3-km buffer increased access to snacks, and, in turn, encouraged the purchase of snack foods. Therefore, access to EDNP food includes a consideration of geographic proximity. Non-poor Nielsen households made an average of only 15.7 and 18.9 trips to purchase snacks and beverages, respectively, in 2010. Thus, because the overall frequencies of purchasing snacks and beverages were not high, it is unlikely that a short distance was the only factor that encouraged impulse buying of snacks in the neighborhood. It is important to know what, if any, other food and non-food items people bought when they purchased snacks. In our companion study [33], we observed that a majority of non-poor households in neighborhoods with many convenience stores acquired fewer fruits than households in neighborhoods with few such stores. This finding, combined with the positive association between convenience store prevalence and snacks purchased by non-poor households, suggests that convenience stores forestalled demand by non-poor households for fresh and nutritious foods by encouraging selection of energy-dense and nutrient-poor foods. Similarly, in a study that included a relatively equal number of people with different ages, race, sex, and educational attainment, Rummo et al. [20] observed that persons who lived in neighborhoods with relatively greater numbers of convenience stores tended to report poorer diet quality than people who lived in neighborhoods with smaller number of such stores.

We found the inverse association between neighborhood convenience store availability and snack purchases among poor households intriguing. If convenience stores contain a basic provision of fresh produce, this fresh produce in the immediate neighborhoods (i.e., 3-km buffer) of poor households may help explain why poor households purchased a low number of snacks. We did not have information for the in-store availability of fresh produce in convenience stores to test this conjecture. Still, our results suggested that convenience stores may significantly influence the consumption patterns of low-income consumers [7]. Future study should examine the differences in food types sold in convenience stores across different income levels.

We observed that non-poor households purchased fewer snacks when they had one supermarket in their neighborhoods compared with non-poor households that did not have any nearby supermarket. However, this observation does not mean that non-poor households purchased fewer snacks because they purchased more “healthy” foods in neighborhood supermarkets. In our companion study [33], we observed a lack of association between neighborhood supermarket availability and fruit and vegetable purchases among 22,448 mostly (i.e., 95%) non-poor households. Interestingly, the effect of the presence of a neighborhood supermarket in discouraging purchase of snacks disappeared when neighborhoods started to have two or more supermarkets. Our results showed that neighborhoods with many supermarkets were weaker in discouraging the purchase of small-sized non-nutritious foods compared with neighborhoods that had one supermarket. Therefore, we conjecture that the food and built environment associated with neighborhoods that had many supermarkets was greatly different from the environment with only one supermarket.

Like other U.S. cross-sectional studies of the purchase of energy-dilute foods such as fresh fruits and vegetables [46], we observed a relative lack of association between neighborhood supermarket availability and snack or beverage acquisition by poor households. The data indicated that being close to supermarkets does not necessarily mean poor households will use those supermarkets to acquire EDNP foods. Our results contradicted results of a study in poor neighborhoods in Pittsburgh, PA [24]; Vaughan et al. observed that full-service supermarkets were the major outlet from which to purchase beverages. Our results complemented previous findings [46,47,48] on the purchase of healthy foods and suggested that the presence of supermarkets in the neighborhood may not necessarily account for an increase in EDNP foods purchased by poor households. We contemplate that the reason supermarkets did not account for large amounts of EDNP purchases was because there were other ubiquitous outlets for poor households to purchase snacks and beverages in (or beyond) the neighborhood, such as small grocery stores, pharmacies, gas-marts, and dollar stores.

It is interesting that we did not observe that convenience stores and supermarkets were associated with beverage purchases (regardless of income level). It seems that the food stores may relate to the purchase of certain type(s) of foods (for example, snacks) but not the other(s) (for example, beverages). Possibly, compared to the locations for purchasing snacks, beverages were purchased from a dispersed range of stores such as warehouse clubs and drug stores within or beyond the immediate neighborhood; these alternatives then led to an insignificant association between immediate neighborhood food and built environment and beverage purchases.

We observed that the regional destination accessibility as measured by employees in 45-min automobile travel time was inversely associated with snacks purchased (b = −0.003, *p* < 0.001) for non-poor households (data not shown). For example, there were an average of 345,686 and 12,256 employees, respectively, in a 45-min automobile travel time for households in New York-Newark-Jersey City, NY-NJ-PA and in Staunton-Waynesboro, VA. Non-poor households in high accessibility regions (for example, New York-Newark-Jersey City) spent 10.0% ((0.003 × 100) × (12,256−345,686)/10,000 = 10.0) less on snacks than non-poor households in low accessibility regions (for example, Staunton-Waynesboro, VA). Other built environmental factors, such as neighborhood street connectivity (b = −0.005, *p* < 0.05), the availability of neighborhood destinations (b = −0.000, *p* < 0.01), and diversity of neighborhood destinations (b = 0.005, *p* < 0.05) were all associated with snack purchases, although the magnitudes were small. The significant results of the broader built environmental factors offered some clues that social environment exerted influences on the purchase of small-size, often non-nutritious foods. For example, increasing the mix of neighborhood destinations such as churches, child care service, and restaurants may change peoples’ needs, goals, and constraints in an unknown way [32] and, in turn, decrease access to snacks. Combined with the identified associations between store availability and snacks and beverages purchased, our findings were consistent with social-ecological frameworks of health in which physical and social environmental factors contribute to food purchasing behaviors.

Because non-poor households purchased more snacks and beverages than poor households (Table 2), future policies of quality-diet should consider all individuals, not only poor persons. As governments search for policies and interventions to improve diet quality, they should give attention to convenience stores that contribute to the purchase of EDNP foods. Local governments should take advantage of the geographic proximity effect that we identified in the present study. Our results support regulation of the number of convenience stores, particularly among non-poor neighborhoods, although we are not aware of any land use policy or regulation that limits their number. Public health advocates recommend that communities use zoning to establish buffers between businesses and places of gathering such as schools and recreational areas [49,50]. We believe a more acceptable approach to shutting down convenience stores would be increasing the provision of healthy foods in convenience stores and encouraging people to purchase more healthy foods therein. For poor neighborhoods, our results suggest that convenience stores need to sell healthier foods [51]. New stocking requirements for authorized Supplemental Nutrition Assistance Program retailers provide small food stores chances to experiment with strategies that facilitate the purchase of healthy foods [52]. Our results support such experiments. Because over seventy percent of households did not have a supermarket in the immediate neighborhood in our sample regardless of the neighborhood’s income level, and the presence of supermarkets did not encourage the purchase of snacks or beverages, it is appropriate to aim policies at introducing a new supermarket in non-poor neighborhoods. However, for neighborhoods that already have a supermarket, opening more supermarkets should be with approached caution until the mechanisms behind our observations are identified more clearly.

Our study had limitations. Factors that we could not have controlled (e.g., skipped reporting of purchases, small expenditures made at convenience stores) likely introduced errors in our estimates of expenditures. Approximately 95% of our sample was non-poor households, a percent that was disproportionately higher than the national average. The substitution of geographic center points for residences might have led to an inaccurate measurement of the food and built environment, particularly for households that were within large ZCTAs. We lacked built environment data for years other than 2010; thus, we were unable to use longitudinal data to address unmeasured confounders such as residential self-selection. Commercial data sources are error prone. We had expected to differentiate whether the beverages were sweetened or unsweetened and include sweetened beverages only in our analysis. However, the data did not enable adequate discrimination. Therefore, we unintentionally included non-sweetened 100% fruit juice and soft drinks, such as nonalcoholic apple cider. Although fruit juices contribute negligible amounts of nutrients that are of concern to most U.S. adults [36], they were not normally treated as nutrition-poor foods. We had intended to compare the purchase of snacks and beverages outside of the specified buffer to that within the neighborhood; however, the Nielsen data did not disclose the exact locations of stores. We did not have data of in-store food types sold; otherwise, we would have had more clues in determining why more convenience stores encouraged poor households to purchase fewer snacks (perhaps convenience stores serving low-income groups served more as stores selling fresh foods rather than less nutritious foods).

## 5. Conclusions

In conclusion, convenience stores were positively and inversely associated with snack expenditure by non-poor and poor households, respectively. Policies and interventions that focus on increasing the provision of healthy foods in convenience stores may be effective for all. However, the underlying mechanism(s) for these behaviors need clarification before the implementation of interventions.

## Figures and Tables

**Table 1:** Descriptive statistics of household demographic factors.

Characteristics	All (*n* = 48,624)	Poor Households ^a^ (*n* = 2292)	Non-Poor Households ^a^ (*n* = 46,332)
Education ****, %			
≤High school or below	23.9	38.0	23.2
>High school	66.1	51.2	66.9
No female head	10.0	10.8	9.9
Race ****, %			
White	82.1	78.6	82.3
Black	10.2	12.8	10.1
Asian	3.1	1.8	3.2
Other	4.6	6.9	4.5
Household size ****, %			
Single member	26.2	38.8	25.5
Two members	41.4	24.1	42.3
Three members	14.3	12.8	14.4
Four + members	18.1	24.3	17.8
Marital status ****, %			
Married	60.1	35.7	61.3
Widowed	7.9	12.9	7.6
Divorced/separated	15.7	27.0	15.1
Single	16.4	24.4	16.0
Children ****, %			
Yes	22.1	30.7	21.6
Number of workers, %			
0	86.8	86.7	86.8
1	10.6	10.0	10.6
2+	3.4	3.3	3.6

^a.^ We defined the poor and non-poor households according to the 2010 federal poverty threshold from the U.S. Census Bureau, which considers the number of adults, the number of related children aged <18 years, and age. Significance refers to chi-square test of difference in categorical characteristics between poor and non-poor households. **** *p* < 0.001; *n*: number of households; IQR: interquartile range. Soicodemographic data were derived from the Nielsen Homescan Consumer Dataset in 2010. Copyright© 2018, The Nielsen Company.

**Table 2:** Descriptive statistics of household food expenditures and the food, built and social environment around a household’s residence.

Characteristics	All (*n* = 48,624)	Poor Households ^a^ (*n* = 2292)	Non-Poor Households ^a^ (*n* = 46,332)
Annual food expenditure, USD, median (IQR)			
Snacks ****	770.5 (1193.2)	598.9 (1075.9)	778.2 (1199.0)
Beverages ****	856.5 (1429.1)	623.1 (1205.1)	868.3 (1436.7)
Number of neighborhood convenience stores ***, count, median (IQR)	3.0 (6.0)	3.0 (7.0)	3.0 (6.0)
Number of neighborhood supermarkets **, %			
0	72.6	71.5	72.7
1	14.6	13.8	14.6
2+	12.8	14.7	12.7
Regional destination accessibility, 10,000 employees in 45-min automobile travel time **, median (IQR)	6.7 (11.2)	5.4 (10.8)	6.7 (11.3)
Availability of neighborhood destinations **, count, median (IQR)	117.0 (309.0)	120.0 (328.0)	117.0 (307.0)
Neighborhood destination diversity **, entropy, mean (SD)	4.4 (2.4)	4.3 (2.5)	4.4 (2.4)
Neighborhood street connectivity **, intersections per square km, median (IQR)	13.1 (19.8)	13.5 (22.3)	13.1 (19.6)
Percent of zero-car households ****, median (IQR)	2.8 (7.6)	3.5 (9.2)	2.8 (7.4)
Percent of population below poverty level ****, median (IQR)	5.8 (8.9)	7.9 (12.1)	5.7 (8.7)
Urbanicity **, %			
Urbanized area	60.2	57.9	60.3
Urban cluster	4.1	4.7	4.0
Non-urban	35.7	37.5	35.6

^a.^ We defined the poor and non-poor households according to the 2010 federal poverty threshold from the U.S. Census Bureau, which considers the number of adults, the number of related children aged <18 years, and age. Significance refers to two-sample Student’s *t*-test of mean’s difference in continuous characteristics or Wilcoxon rank-sum test of median’s difference in continuous characteristics or chi-square test of difference in categorical characteristics between poor and non-poor households. ** *p* < 0.05; *** *p* < 0.01; **** *p* < 0.001; n: number of households. USD: United States dollar; IQR: interquartile range; SD: standard deviation. Food purchase data and household-level soicodemographic data were derived from the Nielsen Homescan Consumer Dataset in 2010. Copyright© 2018, The Nielsen Company.

**Table 3:** Regression results for snacks purchased (log-transformed) by poor and non-poor households (5th–95th percentile, 3-km buffer).

	Poor Households (*n* = 1913 ^a^)			Non-Poor Households (*n* = 40,854 ^a^)		
	B	SE	*p*	B	SE	*p*
**Availability of Convenience Stores, Count**	−0.008	0.004	**0.033**	0.002	0.000	**0.039**
**Availability of Supermarkets, Count**						
**0 (Ref.)**	---	---	---	---	---	---
**1**	0.017	0.059	0.773	−0.027	0.012	**0.025**
**2+**	−0.001	0.073	0.994	−0.026	0.016	0.120

^a.^ We excluded households who purchased extremely low or high values for purchases of snacks, defined here as less than the 5th percentile or greater than the 95th percentile. *n*, number of households; B, coefficient, SE, standard error; Ref., reference category. *P* values in bold indicate statistically significant associations (*p* < 0.05). Food purchase data and household-level soicodemographic data were derived from the Nielsen Homescan Consumer Dataset in 2010. Copyright© 2018, The Nielsen Company.

**Table 4:** Regression results for beverages purchased (log-transformed) by poor and non-poor households (5th–95th percentile, 3-km buffer).

	Poor Households (*n* = 1944 ^a^)			Non-Poor Households (*n* = 41,063 ^a^)		
	B	SE	*p*	B	SE	*p*
**Availability of Convenience Stores, Count**	0.007	0.004	0.101	0.000	0.001	0.821
**Availability of Supermarkets, Count**						
**0 (Ref.)**	---	---	---	---	---	---
**1**	−0.047	0.068	0.491	0.018	0.014	0.187
**2+**	−0.005	0.086	0.951	0.008	0.018	0.654

^a^ We excluded households who purchased extremely low or high values for purchases of beverages, defined here as less than the 5th percentile or greater than the 95th percentile. *n*, number of observations; B, coefficient; SE, standard error; Ref., reference category. *P* values in bold indicate statistically significant associations (*p* < 0.05). Food purchase data and household-level soicodemographic data were derived from the Nielsen Homescan Consumer Dataset in 2010. Copyright© 2018, The Nielsen Company.

## Supplementary Materials

The following are available online at https://www.mdpi.com/1660-4601/17/20/7517/s1, Table S1: Produce type and Nielsen production group module description; Table S2: Regression results for snacks purchased by poor and non-poor households (5th–95th percentile, large ZCTAs excluded, 3-km buffer); Table S3: Regression results for beverages purchased by poor and non-poor households (5th–95th percentile, large ZCTAs excluded, 3-km buffer); Table S4: Regression results for snacks purchased by poor and non-poor households (5th–95th percentile, 5-km buffer); Table S5: Regression results for beverages purchased by poor and non-poor households (5th–95th percentile, 5-km buffer); Figure S1: Percent of poor households at the CBSA level in the United States (2010).
