# Supplementary material for: Association between Neighborhood Food Access, Household Income, and Purchase of Snacks and Beverages in the United States
Source: Int J Environ Res Public Health. 2020 Oct 15;17(20):7517. doi: 10.3390/ijerph17207517 (PMC7602560; doi:10.3390/ijerph17207517)
Supplement: Supplementary file 1 [file ijerph-17-07517-s001.pdf]

Appendix A: measures

Purchase of snacks and beverages

Table S1. Produce type and Nielsen production group module description.

Food type	Department [a]	Product module/brand [a]
Snacks	Dry grocery	snacks - potato chips
		snacks - tortilla chips
		snacks - puffed cheese
		snacks – remaining
		snacks - corn chips
		snacks – meat
		snacks - pork rinds
		snacks - potato sticks
		snacks - health bars & sticks
		popcorn – popped
		popcorn – unpopped
		snacks - caramel corn
		snacks - pretzel
Beverages	Dry grocery	soft drinks - low calorie
		fruit drinks-other container
		soft drinks – carbonated
		soft drinks – powdered
		fruit drinks & juices-cranberry
		fruit drinks-canned
		remaining drinks & shakes-non refriged
		fruit juice - orange - other container
		fruit juice-remaining
		fruit juice – apple
		fruit juice – grape
		fruit juice - lemon/lime
		fruit juice-nectars
		fruit juice - grapefruit - other cont..
		fruit juice-prune
		fruit juice – pineapple
		fruit juice-grapefruit-canned
		fruit juice-orange-canned
		vegetable juice – tomato
	Frozen foods	fruit drinks & mixes - frozen
		fruit drinks & orange – frozen
		fruit juice - remaining - frozen
		fruit juice - orange - frozen
		fruit juice - grapefruit - frozen
		fruit juice - apple - frozen
		fruit juice - grape - frozen
		fruit juice - unconcentrated - frozen

Note: [a] Department, product module, and brand are all Nielsen defined product codes. Food purchase data and household-level soicodemographic data were derived from the Nielsen Homescan Consumer Dataset in 2010. Copyright○C2018, The Nielsen Company.

In calculating the annual expenditure on snacks or beverages by household, we linked the product file to the purchase file using the UPC numbers (variable name 1=upc, variable name 2=upc_ver_uc) as the joint identifying numbers to create a purchase-product file. Upc_ver_uc indicated different versions of upc. We then linked the purchase-product file to the trip file using the trip number (variable name=trip_code_uc) as the joint identifying number to create a trip-purchase-product file. We linked the trip-purchase-product file to the household sociodemographic file using the household number (variable name=household_code) as the joint identifying number to create a household-trip-purchase-product file.

To properly classify self-reported expenditures, we used the departmental category (dry grocery, frozen goods) and the product module description (e.g., snacks, fresh juice, fresh drinks) to

identify snacks or beverages. We then calculated the self-reported expenditures on snacks and beverages (separately) under each standard-UPC products. We finally summed up the total expenditures for all standard-UPC products by household for 2010.

Appendix B: Percent of households under the poverty line

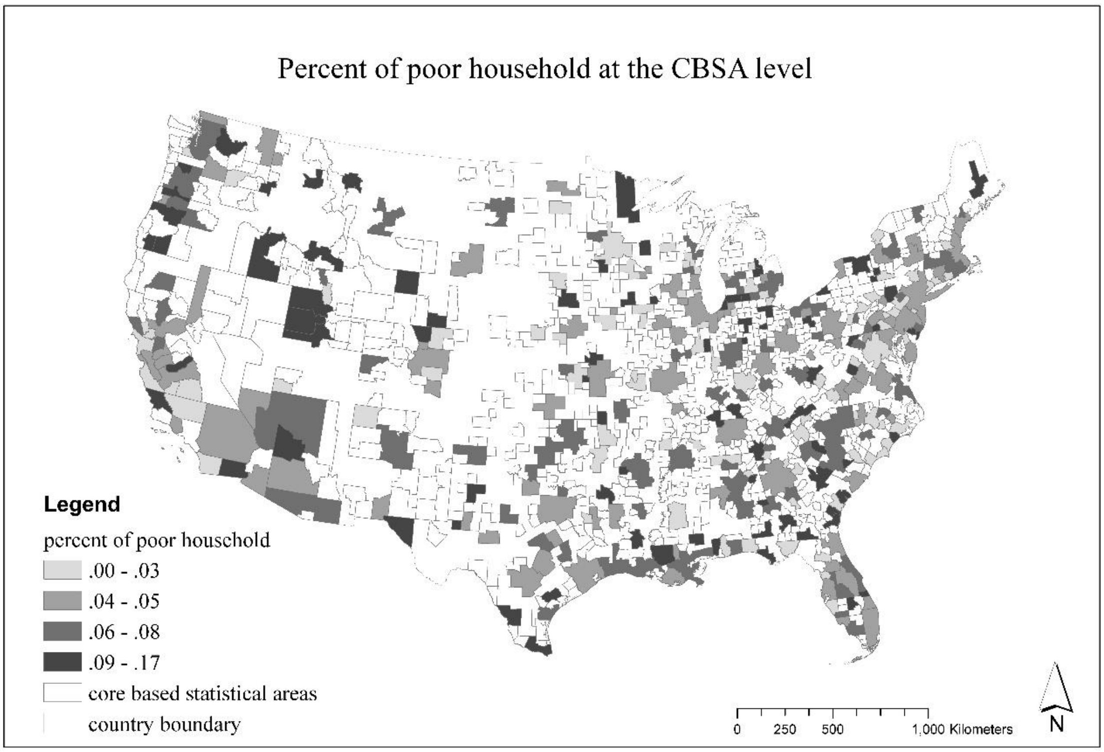

Figure S1. Percent of poor households at the CBSA level in the United States (2010).

Appendix C: data analyses and results

Table S2. Regression results for snacks purchased by poor and non-poor households (large ZCTAs excluded, 3-km buffer).

	**Poor Households (*n* = 1460 [a])**			**Non-Poor Households (*n* = 32,561 [a])**		
	**B**	**SE**	***P***	**B**	**SE**	***P***
Number of convenience stores, count	−0.009	0.004	0.016	−0.002	0.001	0.062
Number of supermarkets, count						
0 (Ref.)	---	---	---	---	---	---
1	0.007	0.065	0.909	−0.028	0.013	0.034
2+	0.025	0.075	0.744	−0.030	0.017	0.070

[a] We excluded households who purchased extremely low or high values for purchases of snacks, defined here as less than the 5th percentile or greater than the 95th percentile. We also excluded who lived in large ZCTAs. P values in bold indicate statistically significant associations ($p < 0.05$). Food purchase data and household-level soicodemographic data were derived from the Nielsen Homescan Consumer Dataset in 2010. Copyright © 2018, the Nielsen Company. *n*, number of observations; CI, confidence interval; Ref., reference category.

Table S3. Regression results for beverages purchased by poor and non-poor households (large ZCTAs excluded, 3-km buffer).

	**Poor households below the poverty line (*n* = 1480 [a])**			**Non-poor households(*n* = 32,806 [a])**		
	**B**	**SE**	***P***	**B**	**SE**	***P***
Number of convenience stores, count	−0.007	0.004	0.085	0.0016	0.001	0.586
Number of supermarkets, count						
0 (Ref.)	---	---	-	---	---	-
1	−0.043	0.074	0.557	0.013	0.014	0.348
2+	−0.011	0.087	0.896	0.005	0.018	0.792

[a] We excluded households who purchased extremely low or high values for purchases of beverages, defined here as less than the 5th percentile or greater than the 95th percentile. We also excluded who lived in large ZCTAs. Food purchase data and household-level soicodemographic data were derived from the Nielsen Homescan Consumer Dataset in 2010. Copyright○C2018, the Nielsen Company. *N*, number of observations; CI, confidence interval; Ref., reference category.

Table S4. Regression results for snacks purchased by poor and non-poor households (5-km buffer).

	**Poor Households** (*n* = 1913 [a])			**Non-Poor Households** (*n* = 40,854 [a])		
	B	SE	*P*	B	SE	P
Number of convenience stores, count	−0.004	0.002	0.011	0.001	0.001	0.140
Number of supermarkets, count						
0 (Ref.)	**-**	**---**	---	---	---	---
1	0.021	0.059	0.719	−0.027	0.005	0.030
2+	−0.000	0.073	0.997	−0.023	0.006	0.159

[a] We excluded households who purchased extremely low or high values for purchases of snacks, defined here as less than the 5th percentile or greater than the 95th percentile. *P* values in bold indicate statistically significant associations ($p < 0.05$). Food purchase data and household-level soicodemographic data were derived from the Nielsen Homescan Consumer Dataset in 2010. Copyright○C2018, the Nielsen Company. *n*, number of observations; CI, confidence interval; Ref., reference category.

Table S5. Regression results for beverages purchased by poor and non-poor households (5-km buffer).

	**Poor households** (*n* = 1944 [a])			**Non-poor households** (*n* = 41,063 [a])		
	**B**	**SE**	***P***	**B**	**SE**	***P***
**Number of convenience stores, count**	0.003	0.002	0.210	0.000-	0.001	0.681
**Number of supermarkets, count**						
**0 (Ref.)**	**---**	**---**	**---**	**---**	**---**	**---**
**1**	0.047	0.062	0.450	0.001	0.013	0.944
**2+**	−0.004	0.072	0.958	−0.005	0.015	0.747

[a] We excluded households who purchased extremely low or high values for purchases of beverages, defined here as less than the 5th percentile or greater than the 95th percentile. Food purchase data and household-level soicodemographic data were derived from the Nielsen Homescan Consumer Dataset in 2010. Copyright○C2018, the Nielsen Company. *n*, number of observations; CI, confidence interval; Ref., reference category.
